# Characteristics and drivers of vegetation productivity sensitivity to increasing CO_2_ at Northern Middle and High Latitudes

**DOI:** 10.1002/ece3.11467

**Published:** 2024-05-23

**Authors:** Yuanfang Chai, Yong Hu

**Affiliations:** ^1^ State Key Laboratory of Earth Surface Processes and Resource Ecology, Faculty of Geographical Science Beijing Normal University Beijing China; ^2^ State Key Laboratory of Loess and Quaternary Geology, Institute of Earth Environment Chinese Academy of Sciences Xi'an China

**Keywords:** CMIP6, nitrogen supply, northern middle and high latitudes, vapor pressure deficit, vegetation productivity sensitivity to CO_2_, water availability

## Abstract

Understanding and accurately predicting how the sensitivity of terrestrial vegetation productivity to rising atmospheric CO_2_ concentration (*β*) is crucial for assessing carbon sink dynamics. However, the temporal characteristics and driving mechanisms of *β* remain uncertain. Here, observational and CMIP6 modeling evidence suggest a decreasing trend in *β* at the Northern Middle and High Latitudes during the historical period of 1982–2015 (−0.082 ± 0.005% 100 ppm^−1^ year^−1^). This decreasing trend is projected to persist until the end of the 21st century (−0.082 ± 0.005% 100 ppm^−1^ year^−1^ under SSP370 and −0.166 ± 0.006% 100 ppm^−1^ year^−1^ under SSP585). The declining *β* indicates a weakening capacity of vegetation to mitigate warming climates, posing challenges for achieving the temperature goals of the Paris Agreement. The rise in vapor pressure deficit (VPD), that triggers stomata closure and weakens photosynthesis, is considered as the dominated factor contributing to the historical and future decline in *β*, accounting for 62.3%–75.2% of the effect. Nutrient availability and water availability contribute 15.7%–21.4% and 8.5%–16.3%, respectively. These findings underscore the significant role of VPD in shaping terrestrial carbon sink dynamics, an aspect that is currently insufficiently considered in many climate and ecological models.

## Graphical abstract

The major contribution of this work is to explore the temporal characteristics of *β* and its driving mechanisms at Northern Middle and High Latitudes. Our results reveal that the *β* in most of land surface has been continuously weakened during past (−0.082 ± 0.005% 100 ppm^−1^ year^−1^, 1982–2015) and future (−0.082 ± 0.005% 100 ppm^−1^ year^−1^ under SSP370 and −0.166 ± 0.006% 100 ppm^−1^ year^−1^ under SSP585) periods, thereby potentially increasing the challenges for achieving the Paris Agreement's long‐term temperature goal. This finding may bring wide‐ranging influences on international and national policy‐making for the weakening capacity of the terrestrial biosphere in absorbing atmospheric CO_2_, especially for countries (e.g., China, European countries, and North America) that highly rely on the CFE to achieve carbon‐neutral goals.

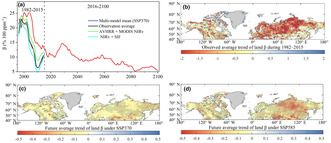

## 1 INTRODUCTION

Increase in atmospheric CO_2_ concentration from fossil fuel combustion and deforestation can enhance plant photosynthetic carbon fixation rates and terrestrial biomass production (Clark et al., 2020; Liu et al., 2022; Morton et al., 2009; Skinner et al., 2018), which is the main driving factor of vegetation greening phenomenon (Piao et al., 2020). Anthropogenic carbon emissions have been partly absorbed by the terrestrial biosphere, making it a critical component of the carbon sink (Xu et al., 2019), especially in the Northern Middle and High Latitudes. It has been reported that the Northern Middle and High Latitudes are generally considered to act as significant land‐based sinks for atmospheric CO_2_.

The sensitivity of vegetation productivity to CO_2_ (*β*), defined as the relative increase in gross primary production (GPP) in response to a 100‐ppm increase in atmospheric CO_2_ concentration (Wang et al., 2020), leads to carbon sequestration in plant biomass. This, in turn, helps mitigate land warming by reducing the rate of increase in atmospheric greenhouse gases (Peñuelas et al., 2017; Reich et al., 2014). Although Wang et al. (2020) have extensively studied the temporal dynamics of *β* at a global scale, the understanding of these dynamics specifically at the Northern Middle and High Latitudes, particularly the underlying mechanisms, remains unclear. The IPCC Fifth and Sixth Assessment Reports have emphasized the high uncertainty surrounding plant physiological responses to increasing CO_2_ concentrations. One key reason for this uncertainty is the influence of various environmental factors, such as vapor pressure deficit (VPD), nutrient availability, and water availability. These factors can significantly impact the magnitude and trends of the plant's physiological responses to CO_2_, making the temporal dynamics of *β* more complex. For instance, some studies have suggested that water availability is the primary limiting factor for plant uptake of atmospheric CO_2_. This limitation can reduce the strength of the terrestrial carbon sink and, in some cases, even lead to temporary carbon sources in terrestrial ecosystems (Jung et al., 2017; Phillips et al., 2009). This temporary reversion from carbon sink into a source has been reported in Amazon forest during 2005 and 2010 (Chai et al., 2021; Dolman & Janssen, 2018), as well as in Europe during 2003 (Ciais et al., 2005), due to droughts events. Another possible driving factor is nutrient limitations, specifically nitrogen and phosphorus supplies, which may restrict CO_2_‐induced biomass enhancement and related carbon sequestration (Oren et al., 2001; Reich et al., 2006, Reich & Hobbie, 2013). Recent studies have also reported the strong limitations of rising VPD on vegetation growth by causing stomatal closure in plants (Grossiord et al., 2020; Helbig et al., 2020; López et al., 2021; Yuan et al., 2019). The highly spatial–temporal heterogeneity in rising VPD, water supply, water consumption, and nutrient availability makes it more challenging to identify the dominated driving factor for the historical *β* changes. Consequently, it becomes difficult for policymakers to implement effective measures to mitigate future global warming.

Terrestrial carbon cycle models, without adequately capturing the concurrent emergence of limiting factors driven by the changing environmental conditions (Anav et al., 2015; Wang et al., 2020), may overestimate *β* when compared with observations. If such underestimation extends into the future, the atmospheric CO_2_ absorbed by ecosystem through *β* will be highly overestimated by the models' projections, leading to an underestimation of the future warming trend at the Northern Middle and High Latitudes. Moreover, the terrestrial carbon uptake may reach a saturation point before the mid‐21st century (Green et al., 2019), where land biomass growth will cease despite the continued increase in atmospheric CO_2_. Approaching or reaching this critical point is concerning as it could accelerate land temperature rise and give rise to a series of ecological, social, and economic issues. A recent study has detected the phenomenon of carbon sink saturation in the world's two most extensive tropical forests, namely the African and Amazonian tropical forests (Hubau et al., 2020). However, the future spatial–temporal dynamics of *β* and its main driving factors remain unexamined at the Northern Middle and High Latitudes. Poor understanding of the responses of future terrestrial ecosystem to rising atmospheric CO_2_ constitutes the largest uncertainty in terrestrial feedbacks to the carbon cycle‐climate system (Reich & Hobbie, 2013) and contributes to the wide range of future climate projections (Ahlström et al., 2017; Campbell et al., 2017).

Here, we conducted a comprehensive analysis of the spatial–temporal dynamics of land *β* at the Northern Middle and High Latitudes during 1982–2015, utilizing three long‐term satellite datasets. To investigate the driving mechanisms behind the changes in *β*, we incorporated observed regional variables such as VPD, nutrient limitations, water supply, and water consumption. To explore the sustainability of the terrestrial carbon sink in the face of land use and climate changes during the future period (2016–2100), we utilized output data from the Earth system models of the latest Sixth Coupled Model Intercomparison Project (CMIP6) to estimate future *β* trends and identify the main driving factors. In this study, we present an integrated picture of past and future characteristics and drivers of *β* at the Northern Middle and High Latitudes.

## 2 DATA SOURCES AND METHODS

### 2.1 Data

#### 2.1.1 Data for estimating the observed *β* during 1982–2015

We estimated the spatio‐temporal dynamics characteristics of observed *β* at each pixel during 1982–2015 using satellite data on GPP, mean CO_2_, maximum air temperature, and VPD (See Section 2.2).

To estimate the GPP at each pixel for each month during 1982–2015, we collected data from various sources as described in the study by Wang et al. (2020) (https://doi.org/10.1126/science.abb7772). These sources include the Advanced Very High Resolution Radiometer (AVHRR NIRV), the fusion of NIRV from AVHRR and Moderate Resolution Imaging Spectroradiometer (AVHRR+MODIS NIRV), and the fusion of NIRV and sun‐induced chlorophyll fluorescence (SIF) (NIRV+SIF).

Additionally, we collected the mean CO_2_ levels based on an air measurement network (https://www.esrl.noaa.gov/gmd/ccgg/trends/) and the monthly maximum air temperature from the Climatic Research Unit (CRU) Time‐Series (TS) version 4.01 (http://data.ceda.ac.uk/badc/cru/data/cru_ts/cru_ts_4.01/). To estimate the monthly VPD during the period of 1982–2015, we collected land surface air temperature and actual vapor pressure from HadCRUT4 Version 4.0.1. The initial monthly climatic data mentioned above has been transformed into the annual average data. Subsequently, we utilized the annual average data to estimate the sensitivity of GPP to inter‐annual changes in CO_2_.

#### 2.1.2 Data for estimating the simulated *β* during 1982–2100

To estimate the simulated spatio‐temporal dynamics characteristics of *β* under the emission scenarios of SSP370 and SSP585, we utilized monthly outputs of GPP, mean CO_2_, maximum air temperature, and VPD for the period of 1982–2100. The monthly outputs of GPP, mean CO_2_, and maximum air temperature were collected from 10 CMIP6 models (Table 1, https://esgf‐node.llnl.gov/projects/cmip6/). To estimate the simulated VPD (See Section 2.2), we collected the monthly outputs of land surface air temperature and relative humidity from the same 10 CMIP6 models. All CMIP6 outputs were gridded to a common spatial resolution of 0.25° × 0.25° latitude‐longitude using the nearest neighbor interpolation method.

**TABLE 1:** Full names of the 10 CMIP6 models used for the monthly data of GPP, temperature, maximum daily temperature, relative humidity during the historical period (1982–2100).

Number	Name	Spatial resolution	Institution
1	BCC‐CSM2‐MR	320 × 160	Beijing Climate Center
2	CanESM5‐CanOE	128 × 64	Canadian Centre for Climate Modeling and Analysis
3	CNRM‐CM6‐1	256 × 128	Centre National de Recherches Meteorologiques
4	CNRM‐ESM2‐1	256 × 128	Centre National de Recherches Meteorologiques
5	INM‐CM4‐8	180 × 120	Institute for Numerical Mathematics
6	INM‐CM5‐0	180 × 120	Institute for Numerical Mathematics
7	IPSL‐CM6A‐LR	144 × 143	Institut Pierre Simon Laplace
8	MIROC‐ES2L	128 × 64	Japan Agency for Marine‐Earth Science and Technology
9	MPI‐ESM1‐2‐LR	192 × 96	Max Planck Institute for Meteorology
10	UKESM1‐0‐LL	192 × 144	Met Office Hadley Centre

#### 2.1.3 Data for investigating the dominated driving factors for the *β* decline during 1982–2015

To investigate the effects of changes in VPD on the observed decline in *β*, we collected monthly data from five observation datasets: HadCRUT4, CFSR, MERRA, ERA‐Interim, and JRA‐55. These datasets cover the period of 1982–2015. For estimating VPD using the HadCRUT4 dataset (See Method), we collected monthly data of land surface air temperature and actual vapor pressure from HadCRUT4 Version 4.0.1 (http://data.ceda.ac.uk/badc/cru/data/cru_ts/cru_ts_4.01/). To estimate VPD using the CFSR, MERRA, ERA‐Interim, and JRA‐55 datasets, we collected monthly data of relative humidity and land surface air temperature. Additionally, we collected monthly outputs of land surface air temperature and relative humidity from the 10 CMIP6 models (Table 1) to estimate simulated VPD during the period of 1982–2015.

To investigate the effects of changes in nutrients, specifically nitrogen (N) and phosphorus (P), on the observed decline in *β*, we collected monthly data on atmospheric N and P depositions during 1997–2013 from the following source: https://figshare.com/s/898e5d6c4f0982674ae3. To investigate the concentrations of N and P in soils, we collected data from a comprehensive survey of publications spanning from the late 1970s to 2012 (https://daac.ornl.gov/cgi‐bin/dsviewer.pl?ds_id=1264). This dataset comprises 3422 data points from 315 papers and includes soil samples primarily collected at depths of 0–15 cm, with some samples collected at depths of 0–30 cm. Additionally, we obtained a map illustrating the patterns of terrestrial nitrogen and phosphorus limitation from a study by Du et al. (2020) (https://doi.org/10.1038/s41561‐019‐0530‐4) (Du et al., 2020).

To investigate the effects of water availability changes on the observed *β* decline, we collected monthly precipitation data from the three precipitation products: HadCRUT4, GPCC, and GPCP, for the period of 1982–2015. The monthly data of the Standardized Precipitation Evapotranspiration index (SPEI) at spatial resolution of 0.5° × 0.5° were provided from SPEIbase v.2.5 for the same period of 1982–2015 (http://digital.csic.es/handle/10261/153475). Additionally, we collected monthly data of soil water content from the CFSR Reanalysis data set (https://rda.ucar.edu/datasets/ds093.1/).

#### 2.1.4 Data for investigating the dominated driving factors for the *β* decline during 2016–2100

To investigate the effects of future VPD on the future *β* decline, we collected monthly projections of land surface air temperature and relative humidity from 10 CMIP6 models for the period of 2016–2100. These projections were used to estimate future VPD at each pixel (Table 1). To investigate the effects of changes in future N in soils on the future *β* decline, we collected monthly projections of N in soils from CMIP6 models during 2016–2100 (https://esgf‐data.dkrz.de/search/cmip6‐dkrz/). To investigate the effects of future changes in water availability on the future *β* decline, we collected monthly projections of precipitation and ET from 10 CMIP6 models. All the projections have been regridded to a common spatial resolution of 0.25° × 0.25° latitude‐longitude.

### 2.2 Method

#### 2.2.1 Method for estimating *β*

A multiple regression approach, which has been widely used by many studies (Piao et al., 2013; Smith et al., 2016; Wang et al., 2020), was applied to calculate *β* at each pixel using 15‐year moving windows for both the past (1982–2015) and future (2016–2100) periods. This approach follows the general linear model (Equation 1).

(1) $$\mathrm{GPP}=\beta \left({\mathrm{CO}}_{2}\right)+a_{1}\left(\mathrm{VPD}\right)+a_{2}\left(T_{\max}\right)+a_{3}+\varepsilon$$

In the multiple regression model, GPP represents the annual gross primary production at each pixel. CO_2_, VPD, and *T*_max_ refer to the annual time series of atmospheric CO_2_ concentration, vapor pressure deficit, and maximum temperature time series at each pixel, respectively. *β*, *a*_1_, *a*_2_, and *a*_3_ are the regression coefficients estimated using maximum likelihood analysis. *ɛ* is the residual error term.

#### 2.2.2 Method for estimating VPD

We estimated the saturated vapor pressure (AVP) using Equations (2), (3), (4) as described in the study by Yuan et al. (2019). In these equations, Tas represents the average land air temperature (°C). Pa is the air pressure (hPa), and Pasl is the air pressure at mean sea level (1013.25 hPa). *H* is the altitude (m).

(2) $$\mathrm{SVP}=6.112\cdot f\cdot e^{\frac{17.67\mathrm{Tas}}{\mathrm{Tas}+243.5}}$$

(3) $$f=1+7\times 10^{-4}+3.46\times 10^{-6}\cdot \mathrm{Pa}$$

(4) $$\mathrm{Pa}=\text{Pasl}\cdot {\left(\frac{\mathrm{Tas}+273.16}{\mathrm{Tas}+273.16+0.0065\cdot H}\right)}^{5.625}$$

The actual vapor pressure (AVP) is calculated using Equation (5), where RH is the land relative humidity (%).

(5) $$\mathrm{AVP}=\frac{\mathrm{RH}}{100}\cdot \mathrm{SVP}$$

To estimate the VPD from the CFSR, MERRA, ERA‐Interim, and JRA‐55 datasets. Equations (2), (3), (4), (5) can be utilized. Additionally, for estimating the VPD from HadCRUT4 during 1982–2015, the AVP can be directly collected from https://data.ceda.ac.uk/badc/cru/data/cru_ts/cru_ts_4.01/. The SVP can estimated using Equations (2), (3), (4).

#### 2.2.3 Physical mechanisms about the effects of rising VPD on terrestrial *β*

Rising VPD typically leads to a reduction in stomatal conductance, which plays a critical role in regulating the photosynthetic uptake of CO_2_. This, in turn, can have negative effects on the terrestrial *β*. Equation (6) demonstrates that assuming transpiration (*T*), ambient (*C*_a_), and inner leaf (*C*_i_) partial pressures of CO_2_ remain unchanged, an increase in VPD directly results in a decrease in the rate of CO_2_ assimilation (*A*).

(6) $$A=\frac{T\cdot C_{a}}{1.6\cdot \mathrm{VPD}}\cdot \left(1-\frac{C_{i}}{C_{a}}\right)$$

#### 2.2.4 Estimating the contributions of potential driving factors to the *β* decline

To explore the relative contribution of each potential driving factor to the *β* decline, we employed multiple regression analysis. This method has been widely used in previous studies (Piao et al., 2013; Smith et al., 2016; Wang et al., 2020). During 1982–2015, we utilized annual data of potential driving factors, including VPD, water availability (Wa, precipitation minus ET) and nutrient availability [depositions of N (Nit) and P (Pho)] with 15‐year moving windows. These factors were used to build multiple regression relationships with the observed *β* at each pixel (Equation 7). It is important to note that the data of nutrient availability only covers the period of 1983–2013. Taking VPD as an example, we obtained the regression coefficient of a1_m_ at grid cell m. By combining the standard deviations of VPD [std(VPD_m_)] and *β* [std(*β*_m_)], we used Equation (8) to estimate the standardized coefficient of VPD (Stc‐VPD_m_). The standardized coefficient represents the relative size of the effects of VPD on the *β* decline, with a higher value indicating a larger effect of VPD on *β* decline. Similarly, we can estimate the standardized coefficients of water availability (Stc‐Wa_m_), N deposition (Stc‐Nit_m_), and P deposition (Stc‐Pho_m_). Using these standardized coefficients, we can estimate the relative contribution of each potential driving factor to the observed *β* decline through Equations (9), (10), (11), (12).

(7) $$\beta_{m}=a1_{m}\left({\mathrm{VPD}}_{m}\right)+a2_{m}\left({\mathrm{Wa}}_{m}\right)+a3_{m}\left({\mathrm{Nit}}_{m}\right)+a4_{m}\left({\mathrm{Pho}}_{m}\right)+\varepsilon$$

(8) $$\mathrm{Stc}‐{\mathrm{VPD}}_{m}=a1_{m}\cdot \frac{\mathrm{std}\left({\mathrm{VPD}}_{m}\right)}{\mathrm{std}\left({\mathrm{CO}}_{2}-{\mathrm{WUE}}_{m}\right)}$$

(9) $$R_{\mathrm{VPD}\_m}=\frac{\left|\mathrm{Stc}‐{\mathrm{VPD}}_{m}\right|}{\left|\mathrm{Stc}‐{\mathrm{VPD}}_{m}\right|+\left|\mathrm{Stc}‐{\mathrm{Wa}}_{m}\right|+\left|\mathrm{Stc}‐{\mathrm{Nit}}_{m}\right|+\left|\mathrm{Stc}‐{\mathrm{Pho}}_{m}\right|}$$

(10) $$R_{\mathrm{Wa}‐m}=\frac{\left|\mathrm{Stc}‐{\mathrm{Wa}}_{m}\right|}{\left|\mathrm{Stc}‐{\mathrm{VPD}}_{m}\right|+\left|\mathrm{Stc}‐{\mathrm{Wa}}_{m}\right|+\left|\mathrm{Stc}‐{\mathrm{Nit}}_{m}\right|+\left|\mathrm{Stc}‐{\mathrm{Pho}}_{m}\right|}$$

(11) $$R_{\mathrm{Nit}‐m}=\frac{\left|\mathrm{Stc}‐{\mathrm{Nit}}_{m}\right|}{\left|\mathrm{Stc}‐{\mathrm{VPD}}_{m}\right|+\left|\mathrm{Stc}‐{\mathrm{Wa}}_{m}\right|+\left|\mathrm{Stc}‐{\mathrm{Nit}}_{m}\right|+\left|\mathrm{Stc}‐{\mathrm{Pho}}_{m}\right|}$$

(12) $$R_{\mathrm{Pho}‐m}=\frac{\left|\mathrm{Stc}‐{\mathrm{Pho}}_{m}\right|}{\left|\mathrm{Stc}‐{\mathrm{VPD}}_{m}\right|+\left|\mathrm{Stc}‐{\mathrm{Wa}}_{m}\right|+\left|\mathrm{Stc}‐{\mathrm{Nit}}_{m}\right|+\left|\mathrm{Stc}‐{\mathrm{Pho}}_{m}\right|}$$

In Equations (7), (8), (9), (10), (11), (12), the subscript m represents the grid cell m; The regression coefficients for each driving factor are denoted as *a*1_m_, *a*2_m_, *a*3_m_, and *a*4_m_; and *ɛ* is the residual error term. Equations (9), (10), (11), (12) provide the estimations of the relative contributions of VPD (*R*_VPD‐m_), water availability (*R*_Wa‐m_), nitrogen deposition (*R*_Nit‐m_), and phosphorus deposition (*R*_Pho‐m_) to the decline in *β* at grid cell m, respectively.

However, it should be noted that the CMIP6 models do not provide outputs for phosphorus deposition for the period of 2016–2100. Consequently, similar to Equations (9), (10), (11), (12), we only estimated the relative contributions of VPD, water availability (precipitation minus ET), and nutrient availability (N in soils) to the predicted *β* decline under the emission scenarios of SSP370 and SSP585.

## 3 RESULTS AND DISCUSSIONS

### 3.1 Vegetation productivity sensitivity to increasing CO_2_ at Northern Middle and High Latitudes

**FIGURE 1:**
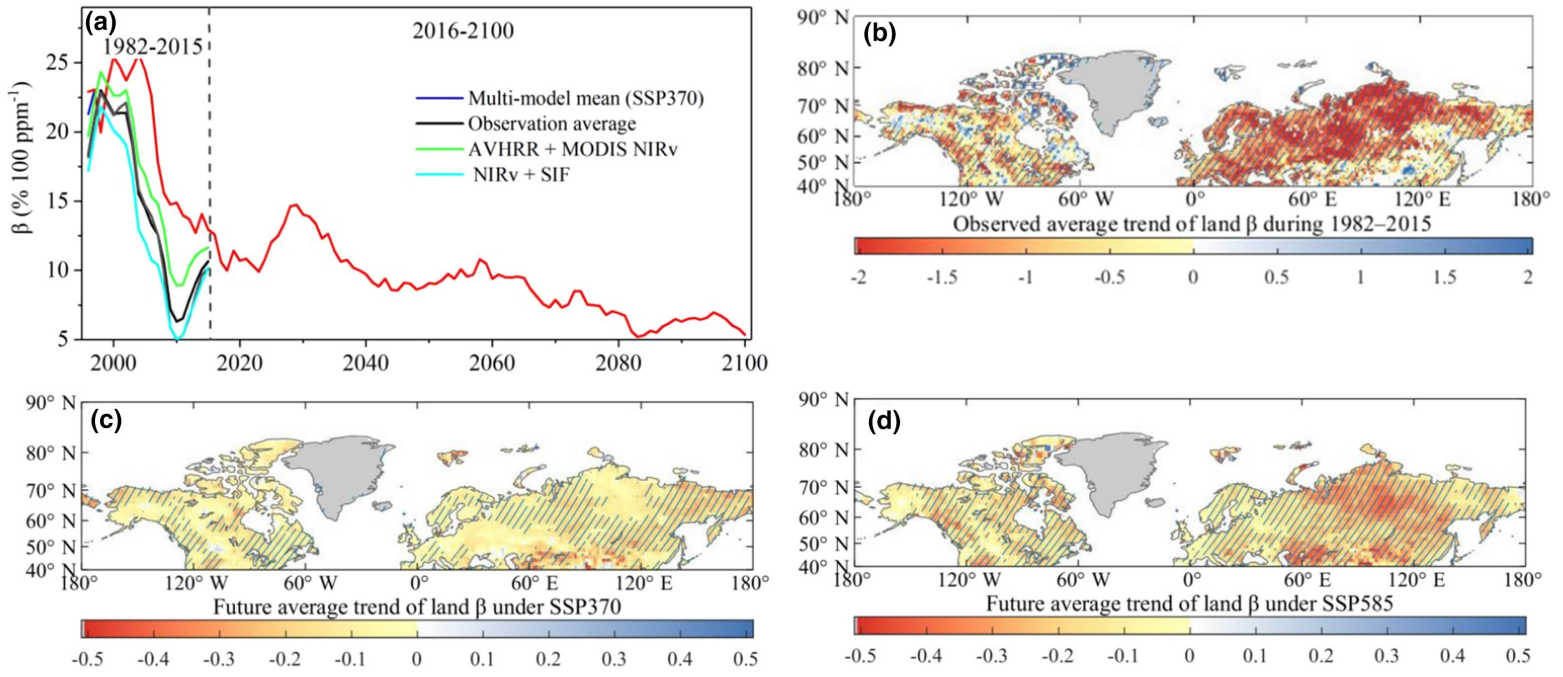
Declining trend of *β* (% 100 ppm^−1^ year^−1^) in the Northern Middle and High Latitudes during 1982–2015. (a) The temporal dynamics of *β* are shown for three satellite GPP proxies using 15‐year moving windows during 1982–2015. Temporal dynamics of *β* for CMIP6 models are presented under the emission scenarios of SSP370 and SSP585 proxies using 15‐year moving windows during 1982–2100. (b) The spatial dynamics of the observation average trend of land *β* during 1982–2015. This is estimated by fitting a linear regression to the *β* time series data in each pixel. The observation average is calculated as the mean value of the *β* trends obtained from AVHRR NIRv, AVHRR+MODIS NIRv, and NIRv+SIF datasets. (c, d) The spatial dynamics of the multi‐model mean trend of land *β* during 2016–2100, under the SSP370 and SSP585 emission scenarios, respectively. The regions with oblique lines represent a significant trend (*p* < .05).

As shown in Figure 1a, all three long‐term satellite data sets (AVHRR NIRv, AVHRR+MODIS NIRv, and NIRv+SIF) exhibit a significant downward trend in *β* during the period of 1982–2015. The average observed decline rate of *β* is −0.89 ± 0.11% 100 ppm^−1^ year^−1^. The decline in *β* is observed to cover approximately 70.9% of the terrestrial area in the Northern Middle and High Latitudes (Figure 1b and Figure S1). This decline can be attributed to both indirect effects on leaf area index (LAI) and direct effect on foliar physiology (Wang et al., 2020). The most direct responses to the decline in *β* are the increases in terrestrial LAI and normalized difference vegetation index (NDVI) (Pan et al., 2018; Yu et al., 2020). However, in contrast to the essentially linear increases in atmospheric CO_2_ concentration, these increases in LAI and NDVI have been progressively weakening according to the GLASS LAI product. This further confirms the observed decline in *β* (Figure S2).

With several improvements, such as increased horizontal and vertical resolutions and new parameterizations for cloud‐radiation interaction and biogeochemical processes (e.g., carbon and nitrogen cycles) (Meehl et al., 2014), all 10 CMIP6 models successfully capture the observed declining trends in *β* during 1982–2015. The multi‐model mean decline rate is estimated to be −0.678 ± 0.094% 100 ppm^−1^ year^−1^. However, it is important to note that most CMIP6 models still exhibit an over‐optimistic view regarding the role of the terrestrial ecosystem in migrating climate warming. They underestimate the declining trend of *β* in 86.2% of the total areas of Northern Middle and High Latitudes. This underestimation may contribute to the inadequate consideration of the limitations imposed by changing environmental conditions in the CMIP6 models (Zhu et al., 2020).

The remarkable performance of CMIP6 models in reproducing historical trends in terrestrial *β* instills confidence in investigating the future spatial–temporal dynamics of *β* based on model projections. Under the emission scenarios of SSP370 and SSP585, all 10 CMIP6 models consistently project a long‐term declining trend in *β* during 2016–2100 (Figure 1c,d). The projected decline in *β* is estimated to be −0.082 ± 0.005% 100 ppm^−1^ year^−1^ for SSP370 and −0.166 ± 0.006% 100 ppm^−1^ year^−1^ for SSP585. The declining *β* is expected to encompass over 90% of the total areas in Northern Middle and High Latitudes (Figure 1c,d). This extensive and prolonged decline in *β* will highly weaken the negative feedback of the terrestrial ecosystem on rising CO_2_ concentration. The underlying driving mechanisms behind this phenomenon need to be comprehensively investigated. Such research is crucial in providing theoretical guidance for policymakers in addressing and mitigating the impacts of future warming climate.

### 3.2 Driving factors for the historical *β* decline

#### 3.2.1 Effects of VPD on the *β* decline

**FIGURE 2:**
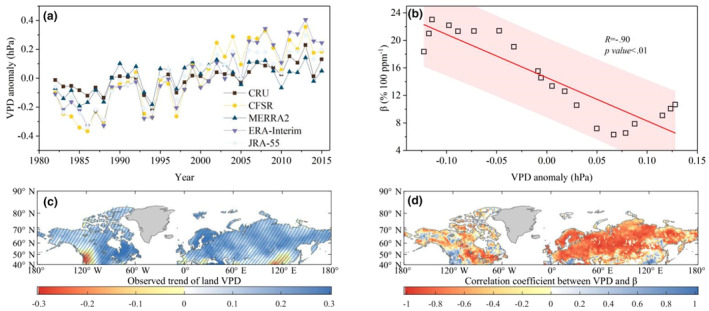
Spatio‐temporal changes in VPD (hPa year^−1^) and their relationships with the decline in *β* at the Northern Middle and High Latitudes during 1982–2015. (a) The temporal dynamics of VPD for the five data sets during 1982–2015. (b) The linear relationships between the observed average VPD anomaly with 15‐year moving windows and the observed average *β* during 1982–2015. (c) Trends in the observed VPD from HadCRUT4 for the period of 1982–2015, estimated by fitting linear regression to the VPD time series data in each pixel. (d) The correlation coefficients (*R*) for the linear regression relationships between the observed VPD from HadCRUT4 and the observed average *β* during 1982–2015. The regions with oblique lines represent a significant trend (*p* < .05).

VPD has been recognized as an important driver of plant structures and functions (Dai, 2013; Grossiord et al., 2020), with the potential to outweigh the effects of changes in precipitation and rising temperature on vegetation productivity (Eamus et al., 2013; Konings et al., 2017). By examining the temporal dynamics of VPD using five observation datasets (HadCRUT4, CFSR, MERRA, ERA‐Interim, and JRA‐55), we found that all five datasets indicate a widespread and significant increase in VPD during 1982–2015 (0.013 ± 0.014 hPa year^−1^, Figure 2a). This increase in VPD is primarily attributed to warming temperatures and drought events (Zhang et al., 2019), covering a large portion of the Earth's land surface (Figure 2c). When the VPD increases, it creates a larger gradient of water vapor concentration between the leaf and the surrounding atmosphere. This increased gradient leads to an accelerated rate of water loss from the leaf through transpiration. In response to prevent excessive water loss and maintain proper hydration, plants have evolved a mechanism to close their stomata. Stomata are small openings present on the surface of leaves that regulate the exchange of gases, including the intake of carbon dioxide for photosynthesis and the release of oxygen and water vapor. When stomata close, the flow of CO_2_ into the leaf is restricted, limiting the availability of this crucial substrate for photosynthesis. Moreover, the higher levels of VPD can also contribute to increased mortality and drive shifts in plant communities (Massmann et al., 2019; McDowell et al., 2020; Sinclair et al., 2017; Will et al., 2013).

The potential negative effects of VPD on *β* are further supported by analyzing the correlation between VPD and *β* using 15‐year moving windows during 1982–2015. In Figure 2b, the VPD data from the five observation‐based datasets exhibit a highly consistent negative correlation with *β* (ǀ*R*ǀ = .9, *p* value < .01). Additionally, the geographic distribution of linear relationships reveals that the tight negative correlations between *β* and VPD span approximately nine‐tenths of the total areas in Northern Middle and High Latitudes (Figure 2d). These strong negative correlations confirm the significant constraint of rising VPD on the decline in *β*. These findings align with recent studies that emphasize the key roles of VPD in vegetation growth (Grossiord et al., 2020; Helbig et al., 2020; López et al., 2021; Yuan et al., 2019). Assuming transpiration, ambient, and inner leaf partial pressures of CO_2_ remain unchanged, Equation (6) suggests that higher VPD directly decreases the rate of CO_2_ assimilation, thereby reducing *β* (Beer et al., 2009; Zhou et al., 2014). This theoretical support further strengthens the understanding of the driving factors behind the decline in *β*.

It should be noted that increasing temperatures have a detrimental effect on the *β*. The highly consistent relationships observed between temperature and the *β*, as well as between VPD and the *β* (Text S1), indicate that the temperature's constraints on the *β* are primarily achieved through the effects of increasing VPD, driven by warming temperatures. The mechanisms are as follows: an increase in VPD driven by rising temperatures can impose water stress on plants and lead to physiological constraints, such as leaf wilting and growth stagnation. These stressful conditions can weaken the plants' response to carbon dioxide, impairing their growth and development, and ultimately reducing the *β*.

The CMIP6 models demonstrate good performance in reproducing the historical increases in VPD under a warming climate during 1982–2015, as evidenced by the high correlation coefficients (*R* = .99) between the observed and simulated average VPD anomalies (Figure S3a). To investigate whether Earth System Models (ESMs) capture the observed constraint of VPD on *β*, linear relationships between VPD and *β* are established using the least squares criterion. Figure S3b shows that the CMIP6 multi‐model mean exhibits a robust negative relationship between VPD and *β* (ǀ*R*ǀ = .86, *p* value < .001). A negative relationship between yearly changes in VPD and GPP suggests that models with higher increases in VPD generally exhibit lower rates of GPP increases (Figure S3b). This provides a strong evidence of the impact of rising VPD on the *β* decline. Poor and positive correlations between *β* and VPD might be attributed to human‐induced greening, including afforestation activities and agricultural activities.

However, the CMIP6 models still underestimate the impact of rising VPD on vegetation growth, as evidenced by the exploration of *β* sensitivity to VPD. The analysis of five observed VPD datasets reveals a significant sensitivity of *β* to VPD during 1982–2015. On average, the observed *β* decreases by 0.629% 100 ppm^−1^ with a 1 Pa increase in VPD. In contrast, most models exhibit a lower negative feedback of terrestrial ecosystem to rising VPD, with a multi‐model mean sensitivity of −0.482% 100 ppm^−1^ Pa^−1^. This underestimated sensitivity indicates that the current ESMs, similar to CMIP5 (Smith et al., 2016), may still underestimate stomatal closure in response to rising VPD. Consequently, there is a tendency for the models to overestimate the *β* on land greening phenomenon when compared to observed estimates (Figure 1a). One possible reason could be Spatial and Temporal Resolution: CMIP6 models generally operate at coarse spatial resolutions, which can limit their ability to capture fine‐scale processes and heterogeneity in the Northern Middle and High Latitudes. Additionally, their temporal resolution may not be sufficient to capture short‐term variability and rapid changes in carbon dynamics.

#### 3.2.2 Effects of nutrient availability on the *β* decline

Field experiments and model simulations have indicated that the response of ecosystems to rising atmospheric CO_2_ concentration is strongly affected by the availability of N and P (Fisher et al., 2012; Fleischer et al., 2019; Schulte‐Uebbing & de Vries, 2018; Zaehle et al., 2010; Zheng et al., 2019). Insufficient N/P deposition can undermine the enhancement of biomass accumulation in response to elevated CO_2_ in several ways (He et al., 2017; Kou‐Giesbrecht & Menge, 2019; Wang et al., 2010; Wieder et al., 2015), including limiting leaf area and light interception, as well as limiting photosynthetic capacity and other aspects of physiological N/P utilization (Reich & Hobbie, 2013). The amplitude of the effects of inadequate N/P supplies on *β* largely depends on local climatic characteristics, vegetation types, soil N/P reserves, and other factors (Cui et al., 2019; Knoepp et al., 2018; Langley & Megonigal, 2010; Reed et al., 2012).

**FIGURE 3:**
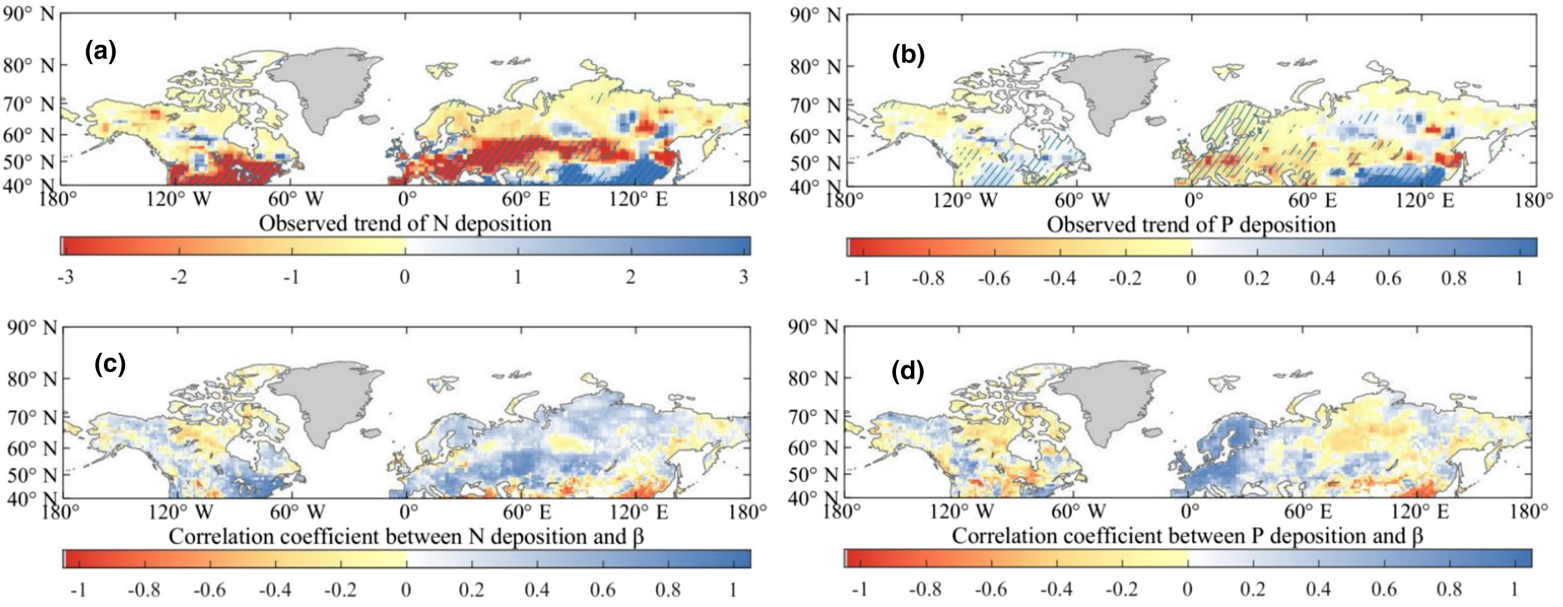
Spatio‐temporal changes in N/P deposition (mg m^2^ year^−1^) and their linear relationships with the decline in *β* at the Northern Middle and High Latitudes during 1982–2015. (a, b) The trends in N and P depositions respectively, using 15‐year moving windows. (c, d) The correlation coefficients (*R*, during 1997–2013) for the linear regression relationships between *β* and N deposition, and between *β* and P deposition, respectively. The regions with oblique lines represent a significant trend (*p* < .05).

If N/P supplies are the main factor contributing to *β* decline, we can expect to observe a similar downward trend between N/P deposition and *β*, which would have negative effects on vegetation growth due to insufficient nutrient supply. Specifically, in the Northern Middle and High Latitudes, particularly in Europe and North America, there has been a continuous decrease in N deposition since the 1990s as a result of policy interventions (Dirnböck et al., 2014; Schmitz et al., 2019; Wamelink et al., 2020) (Figure 3a). This decrease in N deposition has led to a strong positive relationship between N and *β* (Figure 3c). In these regions, the decreased N supplies may be one of the key driving factors contributing to the weakened vegetation greening response to rising CO_2_ (Anav et al., 2015). However, the magnitude of the limitations imposed by decreased N deposition on *β* remains highly uncertain due to the lack of geographical distribution data and long‐term trend characteristics of soil N. Additionally, there is a limited number of ecologically realistic long‐term experiments that investigate the combined effects of atmospheric CO_2_ and N supply (Zaehle et al., 2014, 2015).

Using N deposition in Europe as an example, although N deposition has decreased significantly since the 1990s (Figure 3a), the annual deposition values of N in Europe are the still highest compared to other regions with a decline in *β*. In fact, the annual deposition values may even exceed the N‐saturated stage (>800 mg m^2^ year^−1^) in Europe, which is a critical point where N availability exceeds microbial and plant demands (Law, 2013; Lu et al., 2013). Furthermore, when investigating the N/P concentrations in soils from 315 papers, high concentrations of soil N still can be found in Europe (Figure S4). Hence, despite experiencing several decades of decrease, the high N depositions in these regions at the current level may still be sufficient to support rates of vegetation carbon sequestration and thus may not be the primary cause for the *β* decline.

Importantly, special attention should be given to the high northern latitudes (>55° N), where the vegetation growth is highly limited by N availability. Furthermore, it is crucial to note that the deposition values of N in these regions are low (<200 mg m^2^ year^−1^) (Du et al., 2020). Therefore, the declining trend of N deposition since the 1990s may be associated with the decline *β* in the high northern latitudes due to their strong negative relationship (Figure 3a).

In contrast, the P deposition presents a slight increasing trend (Figure 3b), which is consistent with recent studies (Ackerman et al., 2019; Wang et al., 2017). This indicates that there is a significant between the trends in P deposition and *β*, suggesting that the current variation in N/P characteristics is not the dominated factor contributing to the *β* decline at the Northern Middle and High Latitudes. Furthermore, when plotting average *β* against average P deposition, there is a weak linear relationship between *β* and P (Figure 3d), both based on observations and CMIP6 simulations. This further confirms that the current trend in P deposition variation has little negative feedback on the greening phenomenon.

In summary, our findings provide evidence that changes in N/P deposition are not the primary cause of the *β* decline. However, they may still play an essential role in certain sub‐regions where reduced N/P supplies are observed, particularly depending on the local vegetation types and the N/P reserves in the soils.

#### 3.2.3 Effects of water availability on the *β* decline

**FIGURE 4:**
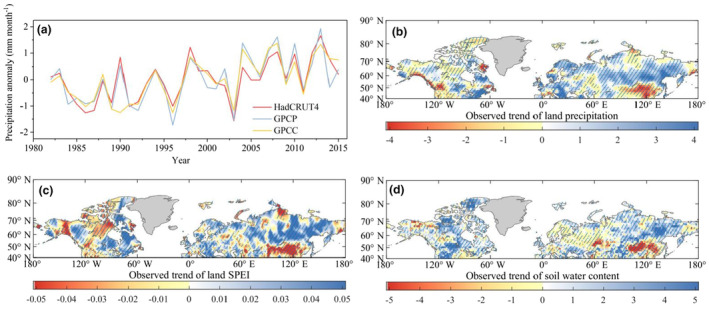
Spatio‐temporal changes in water availability in the Northern Middle and High Latitudes during 1982–2015 using 15‐year moving windows. (a) The changes in annual average daily precipitation during 1982–2015, based on the three precipitation products. (b) The observed trends in precipitation during 1982–2015, using 15‐year moving windows. (c) The trends in SPEI during 1982–2015, using 15‐year moving windows. (d) The trends in soil water content from the CFSR dataset during 1982–2015, using 15‐year moving windows. The regions with oblique lines represent a significant trend (*p* < .05).

Many studies have reported that water availability is another potential limiting factor for terrestrial biological activity (Liu et al., 2018; Papagiannopoulou et al., 2017). Water availability can reduce the benefits of *β* on terrestrial vegetation greening through ecosystem water stress, especially in arid and semi‐arid regions (Ahlström et al., 2015). All the three observed precipitation products present an increasing trend in annual land precipitation across more than half of the land surface in the Northern Middle and High Latitudes during 1982–2015 (0.044 ± 0.012 mm month^−1^ year^−1^, Figure 4a). This suggests that the rising terrestrial water supply has continuously alleviated water stress on vegetation growth, and therefore should not be responsible for the deceleration in land carbon uptake. Furthermore, the highly spatial heterogeneity observed between the trends in *β* and precipitation (Figure 4b) further confirms that changes in precipitation logically are not the dominated factor contributing to the decline in *β*.

The standardized precipitation evapotranspiration index (SPEI) provides a more comprehensive assessment of changes in terrestrial water availability compared to precipitation alone. This is because it incorporates both precipitation and evapotranspiration, which are key components of the water balance in an ecosystem. When analyzing the estimates from SPEIbase v.2.5, we observed that the terrestrial land surface gradually transitioned towards wetter conditions, as indicated by the rising average SPEI for the period of 1982–2015 (Figure 4c). This increase in water availability can benefit vegetation physiological processes rather than leading to a decline in *β*. More than half of the land area in the Northern Middle and High Latitudes experienced an increase in water availability, as evidenced by positive trends in SPEI (Figure 4c). Therefore, the changes in water availability, as reflected in SPEI, are logically not the driving factors for the *β* decline in regions with rising SPEI. Soil water serves as a direct source for the water demands of transpiration and photosynthesis processes (Gu et al., 2019; Liu et al., 2020), and it has effects on the terrestrial vegetation biomass especially in water‐limited ecosystems (Lozano‐Parra et al., 2018). However, similar to precipitation and SPEI trends (Figure 4b,c), observed soil water from CFSR exhibits a slightly rising trend since 1982 (Figure 4d), which is opposite to the trends observed in *β*. Furthermore, vegetation variability in humid regions, especially in the Northern Middle and High Latitudes, is primarily driven by temperature rather than changes in soil water (Liu et al., 2006; Park et al., 2020). Therefore, a slight decrease in soil water may contribute little to the significantly decreased *β*.

Continuous observed evidence suggests that despite the increases in precipitation, SPEI, and soil water that have alleviated the water stress for vegetation growth(Figure 4), there has still been a significant decline in *β* during 1982–2015 (Figure 1a,c). Thereby, the changes in water availability logically are not the primary drivers for the *β* decline. It is important to note that the rising atmospheric CO_2_ concentration tends to reduce leaf conductance of water vapor, resulting in higher leaf water‐use efficiency (Keenan et al., 2013; Soh et al., 2019). This water‐use efficiency can enhance *β* even under conditions of low or decreased soil water (Reich et al., 2014). Consequently, soil water savings resulting from the rising water‐use efficiency could partially or fully offset any constraints imposed by low or decreased water availability on *β*. Consistent with this possibility, studies involving four experiments on *β* and vegetation growth under varying water availability levels have shown no strong dependency on the level of water availability (Andresen et al., 2010; Engel et al., 2009). Therefore, even in regions with decreased water availability, it is still unclear to what extent the decreased water availability contributes to the *β* decline in water‐limited ecosystems.

### 3.3 Driving factors for the future *β* decline

**FIGURE 5:**
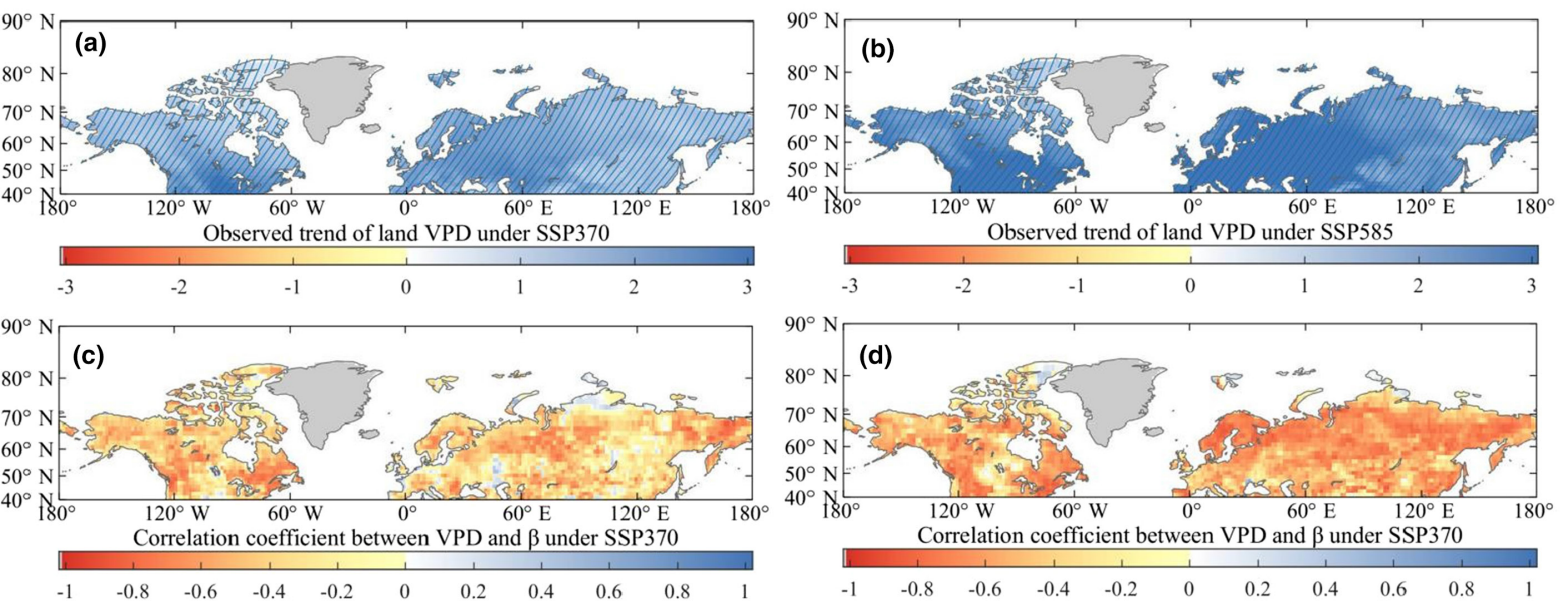
Future spatio‐temporal changes in VPD and their linear relationships with the decline in *β* at the Northern Middle and High Latitudes during 2016–2100. (a, b) The future trends in VPD during the period of 2016–2100 under the SSP370 and SSP585 scenarios, respectively. These trends were estimated by fitting linear regression to the VPD time series data (15‐year moving windows) in each pixel. (c, d) The correlation coefficients (*R*) under the SSP370 and SSP585 scenarios, respectively. These coefficients were estimated by fitting linear regression between the future VPD time series data (15‐year moving windows) and the future *β* time series data (15‐year moving windows) in each pixel. The regions with oblique lines represent a significant trend (*p* < .05).

The projections from CMIP6 models indicate a significant increasing trend in VPD under the emission scenarios of SSP370 (0.0158 ± 0.0003 hPa year^−1^) and SSP585 (0.0267 ± 0.0005 hPa year^−1^) during 2016–2100 (Figure 5a,b). The increasing trend is observed in over nine‐tenths of the land area in the Northern Middle and High Latitudes. The rising VPD will result in vegetation closing their stomata to minimize water losses, consequently weakening the ability of terrestrial ecosystems to act as a land carbon sink through carbon sequestration processes. Strong negative linear relationships (Figure 5c,d) between average annual VPD and average annual *β*, regardless of the emission scenario (SSP370 or SSP585), support the dominant contribution of increasing VPD to the future *β* decline. At a regional scale, the decline in *β* covers a significant portion of the land surface in the Northern Middle and High Latitudes, which is closely associated with the global increase in VPD (Figure 5a,b). The geographical distribution of the strong negative relationships further confirms the substantial constraint imposed by rising VPD on the land *β* (Figure 5c,d).

**FIGURE 6:**
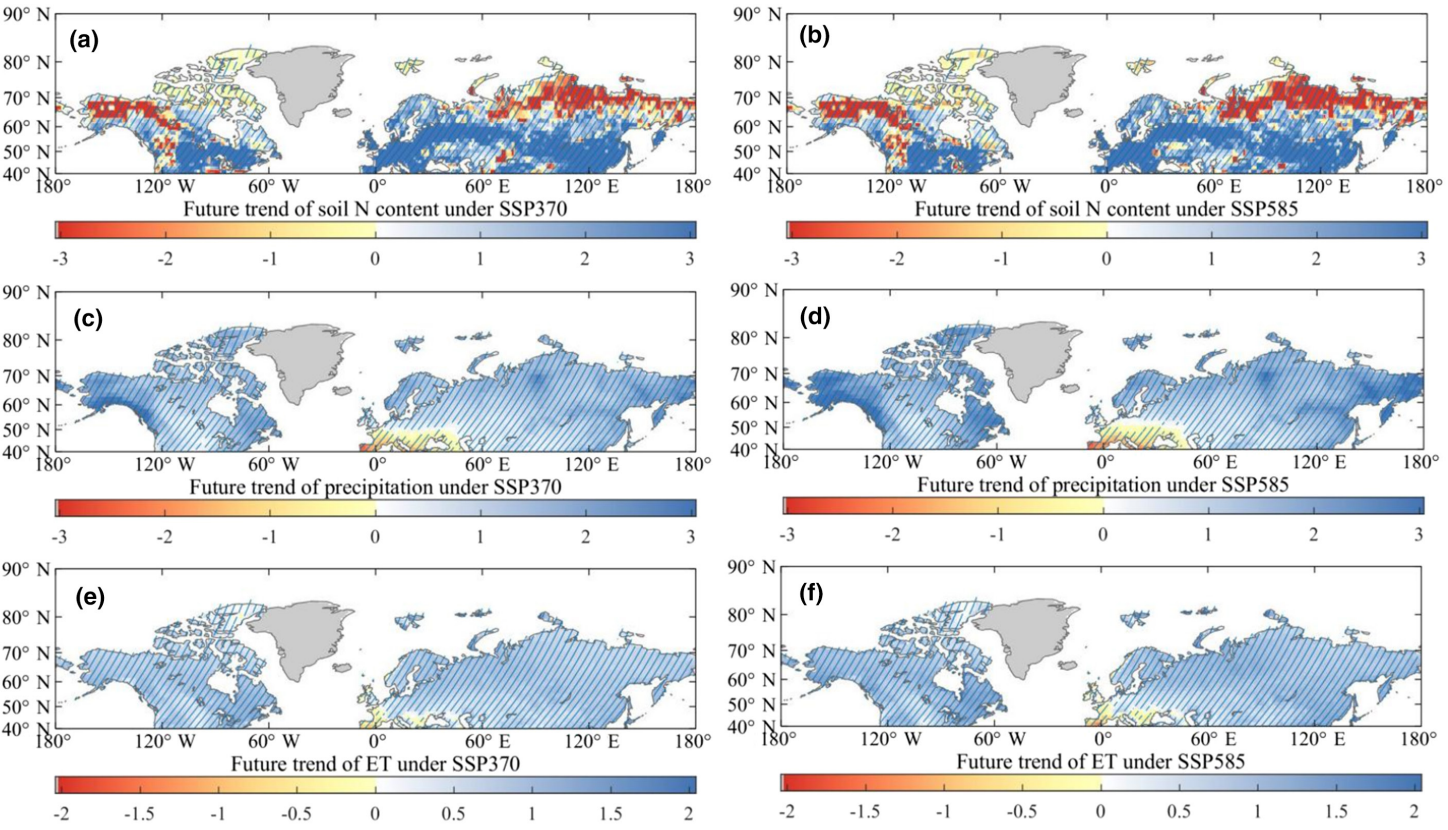
Future spatio‐temporal changes in N in soils, precipitation, and ET at the Northern Middle and High Latitudes during 2016–2100, using 15‐year moving windows. (a, b) The future trends in soil N content during the period of 2016–2100 under the SSP370 and SSP585 scenarios, respectively. (c, d) The trends in future precipitation under the SSP370 and SSP585 scenarios, respectively. (e, f) The trends in future ET under the SSP370 and SSP585 scenarios, respectively. The regions with oblique lines represent a significant trend (*p* < .05).

During the 21st century, it is expected that N deposition over land will increase by a factor of 2.5, primarily due to rising nitrogen emissions from anthropogenic sources (Lamarque et al., 2013). Consequently, the N content in soils, which serves as a direct source of nutrients for vegetation growth, also shows a significant increase trend (Figure 6a,b) for the period of 2016–2100 based on the projections from CMIP6. This continuous rise in soil N content will alleviate nutrient limitations in terrestrial ecosystems and promote vegetation greening (Piao et al., 2015), rather than weakening the rate of biological carbon sequestration (*β* decline). The spatial distributions of changes in soil N content reveal that the majority of the land surface, under both SSP370 (Figure 6a) and SSP585 (Figure 6b), will experience an increase in nutrient availability, even with the concurrent rise in total plant nitrogen uptake. Consequently, the increased nutrient supply outweighs the N consumption for vegetation growth, resulting in enhancement of soil nutrients. Therefore, the increased nutrients in soils should not be considered responsible for the global decline in *β* during 2016–2100.

The atmosphere is expected to reduce its radiative energy under rising temperature, leading to increased longwave emission due to higher temperatures (Previdi, 2010). In order to maintain energy balance, increased precipitation acts as an important compensating process, resulting in increased atmospheric latent heating. All CMIP6 models indicate a clear increasing trend in precipitation under the emission scenarios of SSP370 (Figure 6c) and SSP585 (Figure 6d) during 2016–2100. The future increases in precipitation are widespread and consistent across more than nine‐tenths of the land surface in the Northern Middle and High Latitudes. Certainty, based on the projections from CMIP6 models, future terrestrial total evaporation under SSP370 and SSP585, is also expected to increase due to warming and the phenomenon of vegetation greening (Figure 6e,f). By applying the widely used metric of “precipitation minus evapotranspiration,” we find that the future annual growth rate of water supply from precipitation (Figure 6c,d) is distinctly higher than the increase rate of total evaporation (Figure 6e,f). This will shift the land surface towards wetter conditions. The future continuous increase in water availability will reduce water stress for vegetation growth, instead of impeding rates of carbon sequestration.

## 4 CONTRIBUTIONS OF DRIVING FACTORS ON THE *β* DECLINE

**FIGURE 7:**
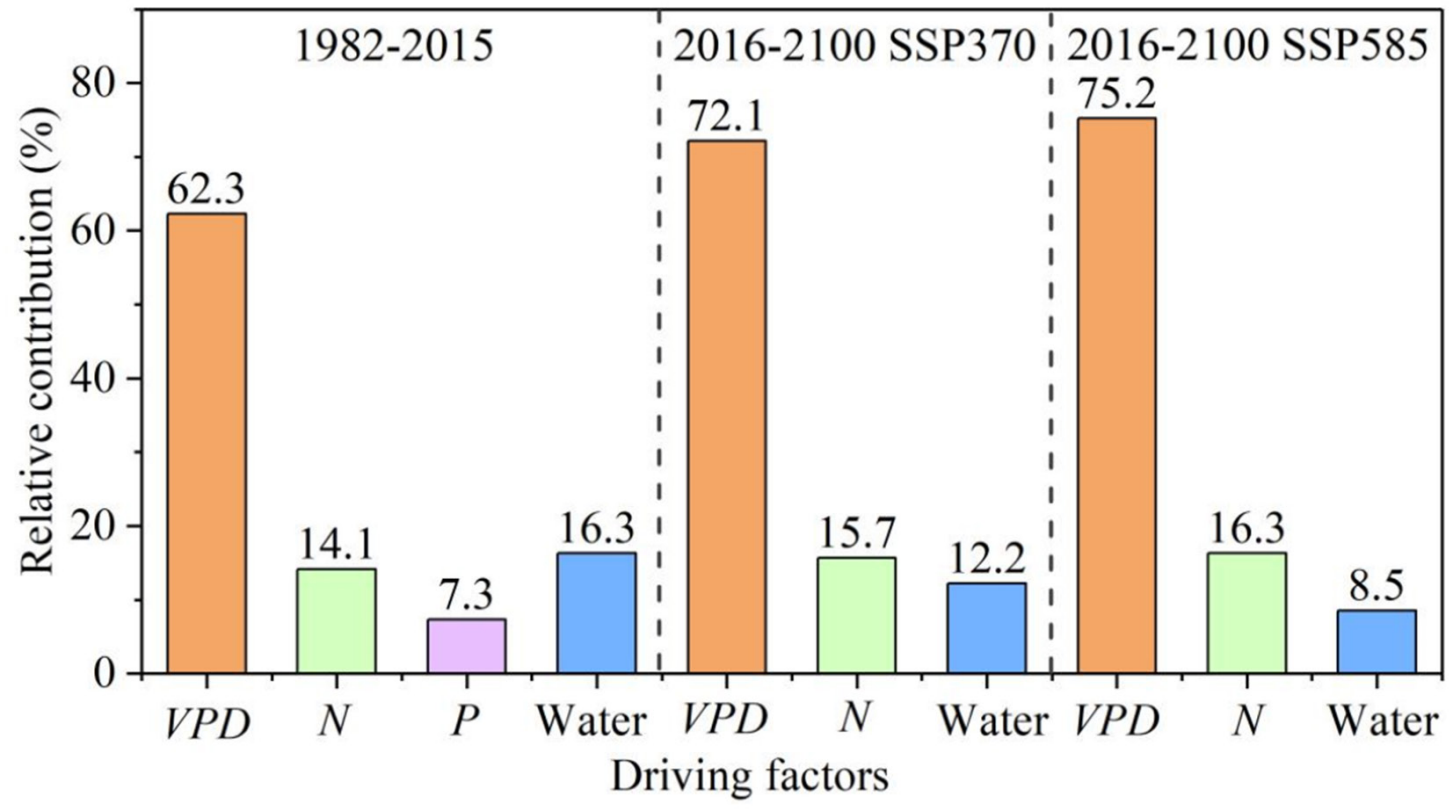
Contribution of each potential driving factor to the past and future *β* decline at the Northern Middle and High Latitudes. During 1982–2015, N deposition and P deposition are considered as the nutrient availability. Precipitation minus ET is used to represent the water availability. During 2016–2100, CMIP6 models lack the outputs of N deposition and P deposition. Therefore, we use N in soils to represent the nutrient availability.

Through a sensitivity analysis, we isolated the contributions of potential driving factors, namely water availability, nutrient availability, and VPD, to the *β* decline. The results of our sensitivity analysis indicate that the observed *β* decline during the period of 1982–2015 is primarily driven by the increase in VPD, accounting for 62.3 ± 15.8% of the decline (Figure 7). On the other hand, limitations in nutrients and water availability contribute smaller proportions of 21.4 ± 8.9% and 16.3 ± 5.7%, respectively. This suggests that VPD plays a dominant role in driving the decline in *β*. Furthermore, our analysis indicates that future increases in VPD will continue to dominate the *β* decline under both the SSP370 (72.1 ± 12.9%) and SSP585 (75.2 ± 14.3%) emission scenarios for the period of 2016–2100. In contrast, nutrients and water availability contribute only 15.7%–16.3% and 8.5%–12.2% (Figure 7), respectively. This sensitivity analysis further emphasizes the significant role of increasing VPD in driving the spatial–temporal dynamics of *β*.

It is important to note that the influence of VPD on the *β* is complex and can interact with other environmental factors. Temperature, in particular, has an overlapping portion on *β* with VPD. Therefore, to gain a better understanding of the specific impact of VPD on the *β*, it is crucial to exclude the influence of temperature. We have conducted a partial correlation analysis to address this issue. The results show that the increase in VPD contributes to 51% of the observed changes in the *β*. In contrast, the increase in temperature only contributes to 21.7%. This supports our findings of the dominant role of VPD in driving the decreased sensitivity of GPP to CO_2_. Humphrey et al. (2021) also show that there is a certain degree of correlation between temperature and VPD, as VPD is calculated based on temperature and relative humidity. After a joint analysis of temperature and VPD effects, they also suggest that VPD has a much larger role than temperature in limiting terrestrial carbon sink through photosynthesis. This further confirms the reliability of our findings.

## 5 CONCLUSION

In this study, observed evidence indicates a decline in *β* at Northern Middle and High Latitudes during 1982–2015, and this decline is projected to persist until 2100 based on the CMIP6 projections under the SSP370 and SSP585 emission scenarios. The widespread reduction in *β* has weakened the capacity of the terrestrial carbon sequestration in migrating warming trend, posing challenges for achieving the long‐term temperature goal of the Paris Agreement.

The primary driver for the decline in *β* at Northern Middle and High Latitudes is considered to be the rising. Increased VPD leads to stomatal closure in plants, reducing their ability to absorb atmospheric CO_2_ and weakening photosynthesis. While observations and simulations suggest that nutrient supply is not the main cause of the *β* decline, reduced N supply, particularly in high northern latitudes, may pose a significant constraint on vegetation growth. On the other hand, the continuous increase in water availability, which shifts the land surface towards wetter conditions, can promote vegetation greening instead of causing a decline in *β*.

Our analysis emphasizes the strong influence of rising VPD on vegetation physiological processes by driving stomata closure. This aspect should be integrated into next‐generation models. Models that do not adequately consider VPD limitations may not accurately capture the magnitudes of the land carbon sink and the warming trends. However, current eco‐physiological, land surface, and crop models often do not account for variations in stomatal conductance in response to rising VPD, overlooking the potential feedbacks between water and carbon cycles driven by stomatal closure under increasing VPD.

## AUTHOR CONTRIBUTIONS

**Yuanfang Chai:** Conceptualization (equal); data curation (lead); formal analysis (lead); funding acquisition (lead); methodology (lead); validation (lead); visualization (lead); writing – original draft (lead). **Yong Hu:** Conceptualization (equal); investigation (equal); supervision (equal); writing – review and editing (lead).

## CONFLICT OF INTEREST STATEMENT

The authors declare no competing interests.

## Supporting information


Appendix S1

## Data Availability

The simulated climate data of CMIP6 are available from https://esgf‐node.llnl.gov/projects/cmip6/. The observed temperature data are provided from http://www.cru.uea.ac.uk/ (HadCRUT4 data set). The observed temperature is available from http://www.cru.uea.ac.uk/ (HadCRUT4 data set). The observed precipitation is available from https://climatedataguide.ucar.edu/climate‐data/gpcc‐global‐precipitation‐climatology‐centre (GPCC data set).
