# Supplementary material for: Characteristics and drivers of vegetation productivity sensitivity to increasing CO2 at Northern Middle and High Latitudes
Source: Ecol Evol. 2024 May 23;14(5):e11467. doi: 10.1002/ece3.11467 (PMC11116762; doi:10.1002/ece3.11467)
Supplement: Supplementary file 1 — Appendix S1 [file ECE3-14-e11467-s001.docx]

**Supplementary Materials for**

**Title: Characteristics and drivers of vegetation productivity sensitivity to increasing CO_2_ at Northern Middle and High Latitudes**

**Authors:** Yuanfang Chai^1^, Yong Hu^2^*

1 State Key Laboratory of Earth Surface Processes and Resource Ecology, Faculty of Geographical Science, Beijing Normal University, Beijing 100875, People’s Republic of China.

2 State Key Laboratory of Loess and Quaternary Geology, Institute of Earth Environment, Chinese Academy of Sciences, Xi’an, 710061, China

Corresponding author: Yong Hu [(huyong@ieecas.cn)](mailto:(huyong@ieecas.cn))

**Supplementary Text S1 Temperature constraining on β**

The influence of temperature on the β can be explained through the following aspects: Photosynthetic efficiency: Temperature has a effect on the efficiency of plant photosynthesis. At higher temperatures, the rate of photosynthesis may increase, thereby increasing the plant's demand for CO_2_. Therefore, the β may be more pronounced under warm climatic conditions.Transpiration: Plant physiological responses: Temperature influences plant physiological responses and growth cycles. Under high-temperature conditions, certain plants may experience heat stress, leading to growth limitations or adverse effects, thereby reducing the β. Temperature also affects plant transpiration. Higher temperatures can lead to increased transpiration in plants, resulting in greater water loss. If water supply is limited, plants may not be able to fully utilize the increased CO_2_, thereby weakening the β.

Based on the observed temperature data from HadCRUT4 (Supplementary Fig.6a), it has been found that temperature exhibits a significant negative correlation with the β. As shown in Supplementary Fig.6b, there is an increasing trend in observed temperatures across the entirety of the Northern Middle and High Latitudes. This results in a negative relationship between temperature and the β spanning approximately nine-tenths of the total area of the Northern Middle and High Latitudes (Supplementary Fig.6c).

**
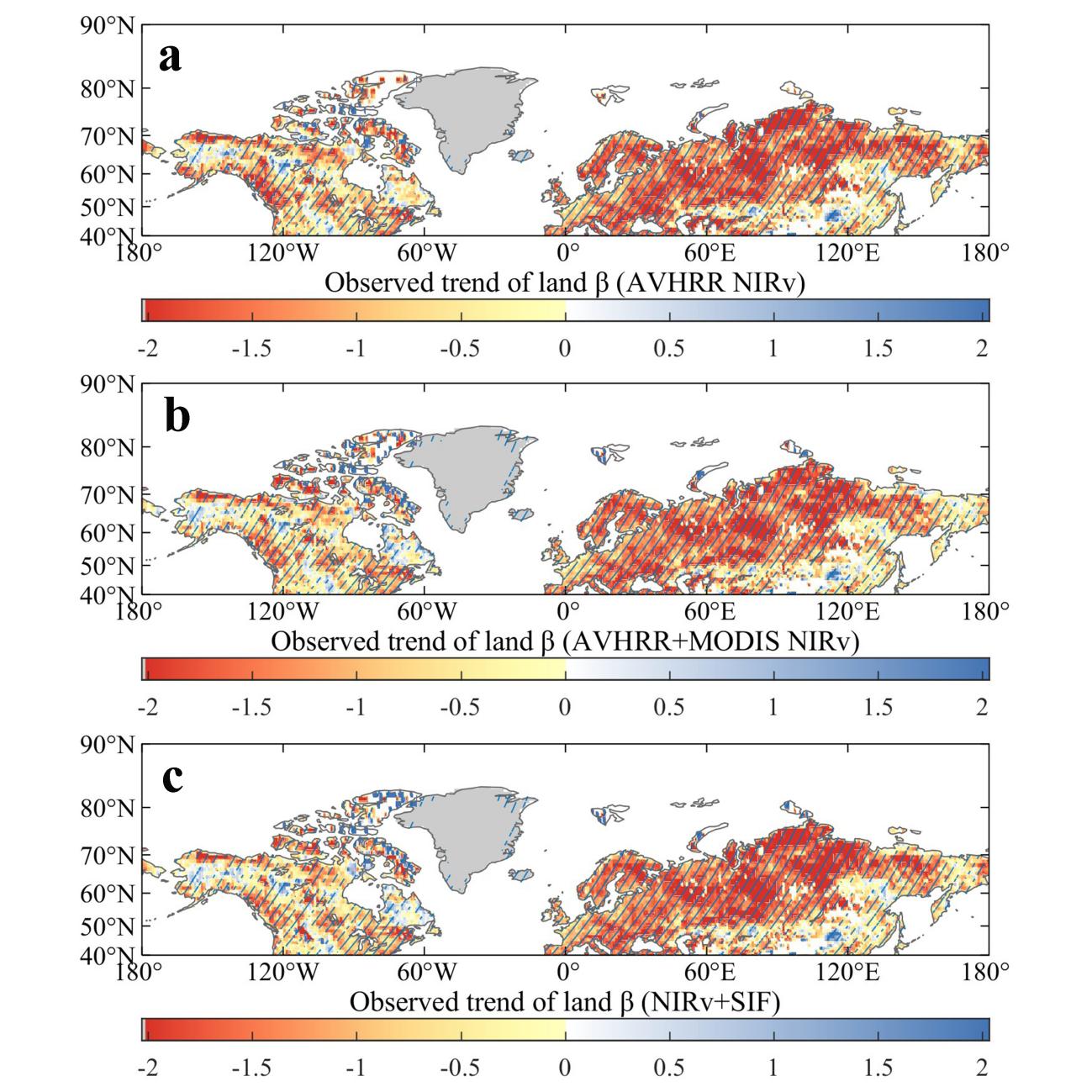
**

**Supplementary Fig. 1** **Spatial dynamics of the trends of land β during 1982**–**2015.** The trends of β are estimated by fitting linear regression to the β time series data in each pixel. (**a**), (**b**) and (**c**) are the trends from AVHRR NIRv, AVHRR+MODIS NIRv and NIRv+SIF data sets, respectively. The regions with oblique lines represent a significant trend (*p*<0.05).


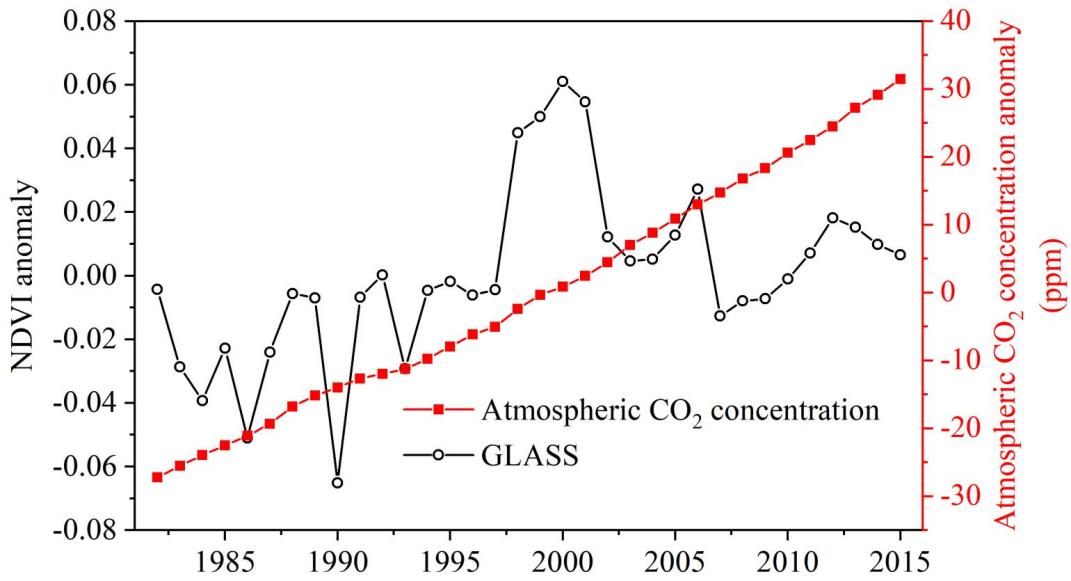


**Supplementary Fig.2 Changes in satellite-derived vegetation indices and atmospheric CO_2_ concentration**. Data of LAI is collected from GLASS data set.


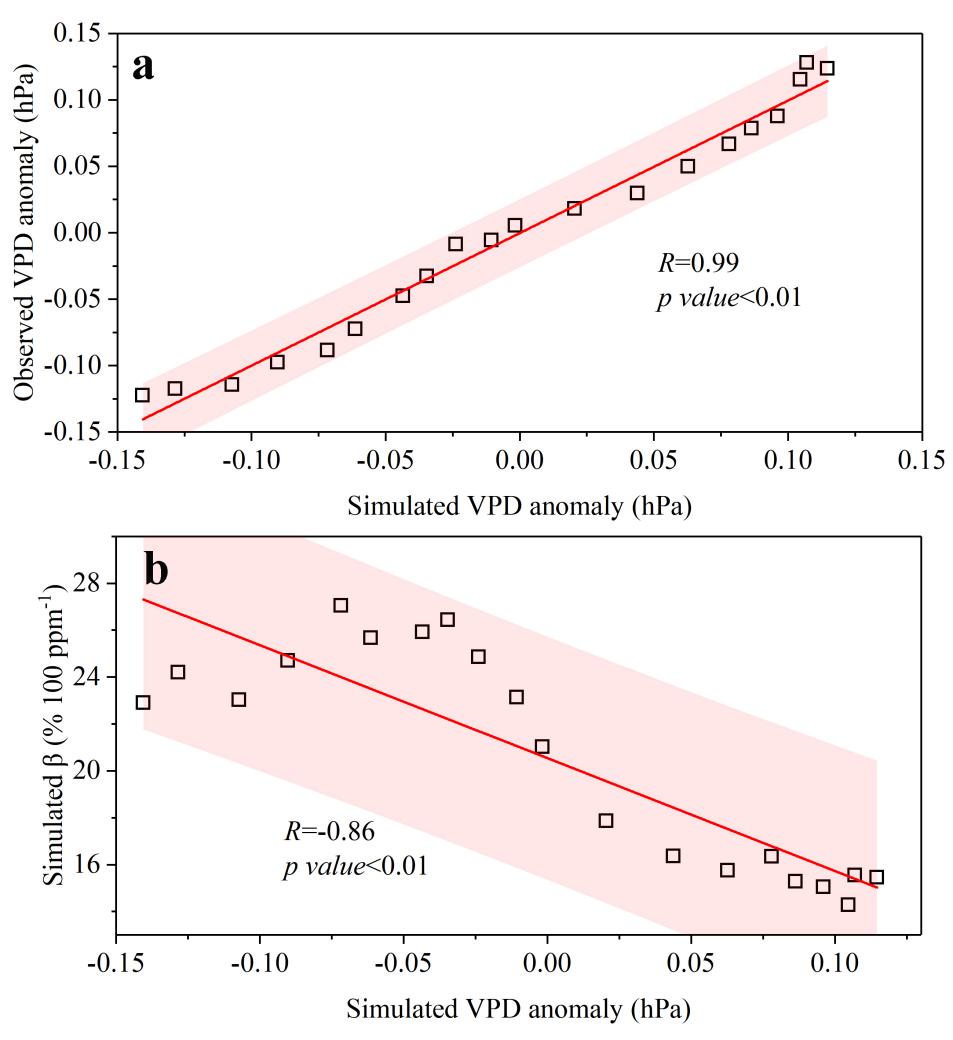


**Supplementary Fig.3 Relationships between simulated VPD and observed VPD (a), and between simulated VPD and simulated β (b) at Northern Middle and High Latitudes.**

**
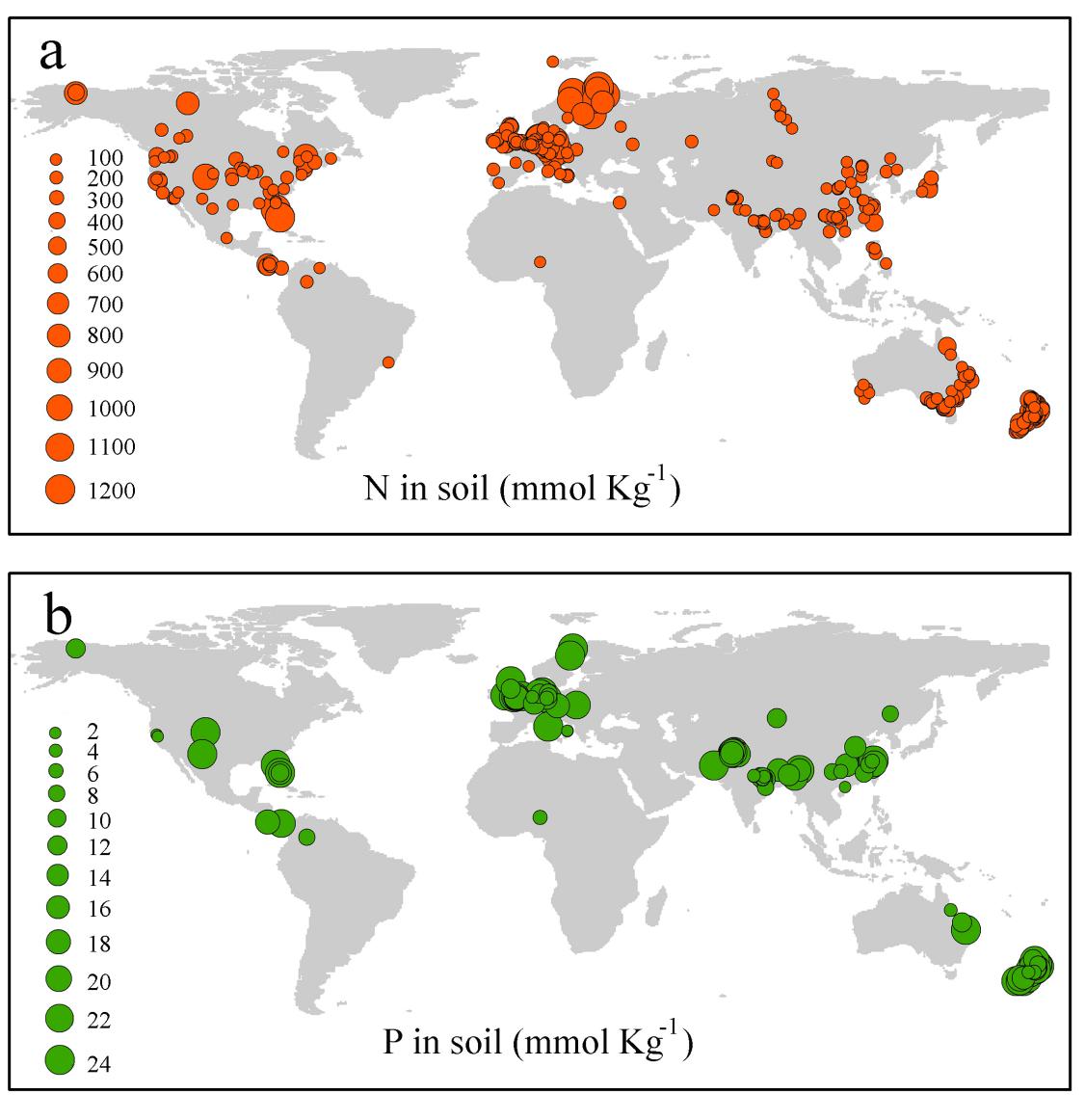
**

**Supplementary Fig.4 Nutrients in soils based on the data from 315 papers.** (**a**) and (**b**) are the N and P in soils, respectively.


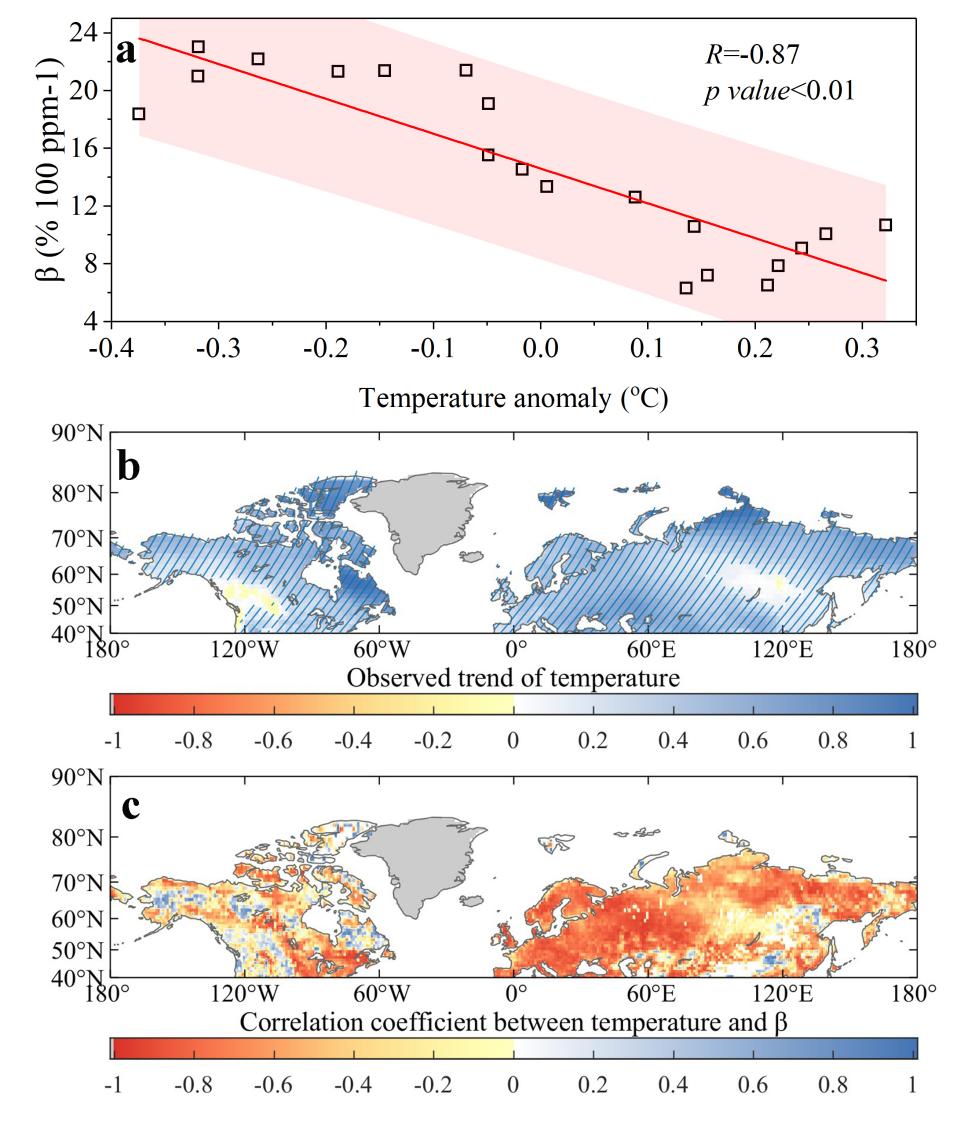


**Supplementary Fig.5** **Spatio-temporal changes in temperature and its relations with β decline at Northern Middle and High Latitudes during 1982**–**2015.** (**a**) is the linear relationships between the observation average temperature anomaly with 15-year moving windows and the observation average β during 1982–2015. (**b**) is trends in the observed temperature from HadCRUT4 for the period of 1982–2015, estimating by fitting linear regression to the temperature time series data in each pixel. (**c**) is the correlation coefficients (R) for the linear regression relationships between the observed temperature from HadCRUT4 and the observation average β during 1982–2015. The regions with oblique lines represent a significant trend (*p*<0.05).
